# Basophils beyond allergic and parasitic diseases

**DOI:** 10.3389/fimmu.2023.1190034

**Published:** 2023-05-02

**Authors:** Remo Poto, Stefania Loffredo, Gianni Marone, Antonio Di Salvatore, Amato de Paulis, John T. Schroeder, Gilda Varricchi

**Affiliations:** ^1^ Department of Translational Medical Sciences, University of Naples Federico II, Naples, Italy; ^2^ World Allergy Organization (WAO), Center of Excellence (CoE), Naples, Italy; ^3^ Center for Basic and Clinical Immunology Research (CISI), University of Naples Federico II, Naples, Italy; ^4^ Institute of Experimental Endocrinology and Oncology “G. Salvatore”, National Research Council (CNR), Naples, Italy; ^5^ Division of Allergy and Clinical Immunology, The Johns Hopkins University School of Medicine, Baltimore, MD, United States

**Keywords:** alarmins, allergy, autoimmunity, basophil, cancer, COVID-19, myocardial infarction

## Abstract

Basophils bind IgE *via* FcεRI-αβγ_2,_ which they uniquely share only with mast cells. In doing so, they can rapidly release mediators that are hallmark of allergic disease. This fundamental similarity, along with some morphological features shared by the two cell types, has long brought into question the biological significance that basophils mediate beyond that of mast cells. Unlike mast cells, which mature and reside in tissues, basophils are released into circulation from the bone marrow (constituting 1% of leukocytes), only to infiltrate tissues under specific inflammatory conditions. Evidence is emerging that basophils mediate non-redundant roles in allergic disease and, unsuspectingly, are implicated in a variety of other pathologies [e.g., myocardial infarction, autoimmunity, chronic obstructive pulmonary disease, fibrosis, cancer, etc.]. Recent findings strengthen the notion that these cells mediate protection from parasitic infections, whereas related studies implicate basophils promoting wound healing. Central to these functions is the substantial evidence that human and mouse basophils are increasingly implicated as important sources of IL-4 and IL-13. Nonetheless, much remains unclear regarding the role of basophils in pathology *vs.* homeostasis. In this review, we discuss the dichotomous (protective and/or harmful) roles of basophils in a wide spectrum of non-allergic disorders.

## 1 Basic concepts of basophils

Basophils are rare blood cells, accounting for 1% or less of the circulating leukocytes-a feature evident both in humans and mice. Basophils share several morphological and functional characteristics with tissue-resident mast cells. Most recognized are the cytoplasmic granules that each cell possesses and that stain so predominantly with basic stains. Phenotypically, both cell types s uniquely express the αβγ_2_ structure of the high-affinity receptor (FcεRI) for IgE, which enables both cells to rapidly release pre-formed histamine and newly generated cysteinyl leukotriene C_4_ (LTC_4_), upon encountering relevant allergen (1, 2). Accordingly, basophils were initially viewed, incorrectly, as blood-circulating mast cells, which prompted the notion of using them as surrogates to study tissue mast cells, which proved far more difficult to obtain (2). However, it is now widely accepted that basophils and mast cells profoundly differ in several fundamental aspects (3). For example, the lifespan of basophils (~days) is much shorter than the months estimated for mast cells (4). Transcriptionally, basophils are more closely related to eosinophils than mast cells (5, 6). These differences (among many more discussed elsewhere (7) suggest that basophils have unique pathophysiological roles different from those of mast cells.

IL-3 is central to the growth, differentiation, priming, and overall activation of both human and mouse basophils (8, 9). It does so by binding, with high-affinity, to the α subunit of its receptor (IL-3Rα/CD123) highly expressed on basophils (10). Many cell types are implicated in producing the IL-3 that impacts basophil development and function, including T cells (11, 12), B cells (13), human eosinophils and neutrophils (14), but also mast cells and even basophils (15, 16). Although the IL-3 receptor is highly expressed on basophils (17–28), mice incapable of producing IL-3 and/or deficient in IL-3Rα/CD123 reportedly develop all blood lineages, including basophils and mast cells (29–31). In this regard, thymic stromal lymphopoietin (TSLP) is also reported to regulate mouse basophil development (32, 33) and activation (9) *in vivo* and may therefore represent an important early growth factor for these cells. In contrast, numerous studies show that IL-3 is quite sufficient in promoting the *in vitro* growth of functional human and mouse basophil-like cells from progenitors. TSLP is reported to activate human basophils from asthmatic subjects by promoting histamine release and cytokine secretion, along with inducing cell surface expression of CD203c and IL-3Rα (34). In contrast, several other investigators have since reported that TSLP does not activate human basophils isolated from healthy subjects or allergic patients (9, 10, 35). In light of the latter findings, TSLP may have very different effects on human *versus* mouse basophils (9). Finally, IL-3 is well known for its capacity to mediate synergistic (or priming) effects when combined with a diverse array of co-stimuli (9, 36–40).

It has been shown in mice that basophils originate from hematopoietic stem cells (HSCs) in the bone marrow (41, 42). So-called granulocyte-macrophage progenitors (GMPs), which develop later than the HSCs giving rise to most of the common myeloid progenitors, are thought to be the relevant basophil progenitors (BaPs (43). Common basophil-mast cell progenitors are also present in the spleen (43, 44). Single-cell transcriptomic analyses have highlighted the differentiation pathways of various cell lineages in mice (45–47). Single-cell culture of mouse bone marrow progenitors generated FcϵRI^+^ basophils and erythroid cells (48). The erythroid trajectory is close to that of basophils/mast cells, both in mice (49) and humans (50–53). Human CD131^+^ CMP progenitors in the bone marrow can differentiate into basophil/mast cell/eosinophil and erythroid/megakaryocyte populations (51). Likewise, studies of human bone marrow cells using single-cell transcriptome analysis found the basophil trajectory to be more linked with that of the megakaryocyte and erythroid lineages, rather than those of granulocytes/monocytes (52). It is likely that the differentiation pathways of basophils and mast cells are more closely linked to those of the erythroid/megakaryocyte lineages, rather than to granulocytes/monocytes, both in mice and humans.

Several analytical tools for the study of mouse basophil biology have been developed in recent years. In particular, the use of antibodies capable of depleting basophils *in vivo* (54, 55) as well as mice that are genetically altered to be deficient of basophils (56–61), which includes reporter mouse models (58, 61), and basophil-specific Cre-expressing mice (58, 62, 63). The results obtained with these different models have demonstrated non-redundant roles of basophils in experimental Th2-type inflammation, comprising certain aspects of various allergic responses (3, 64, 65). Likewise, these models have substantiated the long-held belief that basophils help mediate immunity against parasitic infections (66–69). Some of these analytical tools have been employed to evaluate the role of mouse basophils in myocardial infarction (MI) (70), renal fibrosis (71), cancer (72–75), autoimmune disorders (76, 77), and chronic obstructive pulmonary disease (COPD) (62). **Table 1** lists the antibody-mediated and genetic models for analyzing the *in vivo* contribution of mouse basophils in various pathophysiological conditions.

**Table 1:** Antibody-mediated and genetic depletion models for the *in vivo* study of basophils in different pathological conditions.

Methods to deplete basophils	Examined pathological conditions	References
Antibody-mediated		
Monoclonal antibody (mAb) anti-FcϵRI (MAR-1)	IgE-mediated chronic allergic dermatitis (IgE-CAI)	(54)
mAb anti-CD200R3 (Ba103)	Description of the mAb	(78)
mAb MAR-1	Allergic inflammation	(79)
mAb MAR-1	Myocardial infarction (MI)	(70)
mAb anti-CD2003 (Ba103)	Emphysema	(62)
mAb MAR-1	Kidney fibrosis	(71)
Genetically engineered mice		
*Mcpt8^Cre^* mice	*N. brasiliensis* infection IgE-CAI Systemic anaphylaxis	(57)
*Mcpt8^DTR^* mice	Tick-borne disease	(56)
*Runx1*	IgE-CAI *Strongyloides* infection	(59)
BasTRECK	IgE-CAI	(59)
BasoDTR mice	IgE-CAI	(60)
Basoph8xiDTR mice	Skin allergic inflammation	(61)
Mcpt8^Cre^/DTR mice	Kidney fibrosis	(71)
*Mcpt8* ^DTR^ mice	Emphysema	(62)
*Mcpt8^iCreERT2^Stim1^fl/fl^*	IgE-CAI	(63)
Mcpt8^Cre^ mice	MI	(70)
CT-M8 mice	Systemic Lupus Erythematous	(80)

DTR, diphtheria toxin receptor; IgE-CAI, IgE-mediated chronic allergic dermatitis; mAb, monoclonal antibody; MI, myocardial infarction.

Several outstanding reviews have discussed the roles of mouse and human basophils in allergic disorders (1, 64, 74, 81, 82) and parasitic infections (66–68). Increasing evidences indicate that basophils also play relevant roles in several other types of responses, including autoimmunity (83, 84), myocardial infarction (70), fibrosis (70, 71, 85), cancer (86–88), and COVID-19 (89). In this review, we discuss the recent basophil contribution to the pathogenesis of several non-allergic inflammatory diseases.

## 2 Basophils in myocardial infarction

Myocardial infarction (MI) occurs when coronary arteries that supply oxygen and nutrients to the heart become obstructed by atherosclerotic arterial walls (90). The consequence is an ischemic injury that mobilizes a repertoire of innate and adaptive immune cells (91, 92). Shortly, after ischemic occurs, resident cardiac mast cells release their preformed mediators (93), resident macrophages and cardiomyocytes produce cytokines and chemokines (94, 95), fibroblasts release growth factors (96) and endothelial cells are activated. These events typically cause an influx of various immune cells, including neutrophils, monocytes, macrophages (92, 97), and mast cells (98, 99).

The inflammatory response following MI deeply affects subsequent cardiac remodeling and fibrosis (100, 101). The composition of immune cell types identified in the infarcted myocardium consists mostly of macrophages, monocytes, neutrophils, DCs, B and T cells, and NK cells (70, 97). Using a mouse model, Sicklinger and coworkers demonstrated that basophils infiltrate infarcted hearts, reaching a peak between days 3 and 7 and reverting to baseline on day 14 (70). The administration of the monoclonal antibody (mAb) anti-FcϵRI (MAR-1) depleted basophils in the heart, peripheral blood, and spleen. In contrast, mast cells and a subset of DCs expressing FcϵRI were not altered following MAR-1 administration. Depletion of basophils reduced left ventricular ejection fraction 4 weeks after MI and increased heart weight compared to control. Moreover, basophil-depleted mice showed reduced scar thickness.

**Figure 1:**
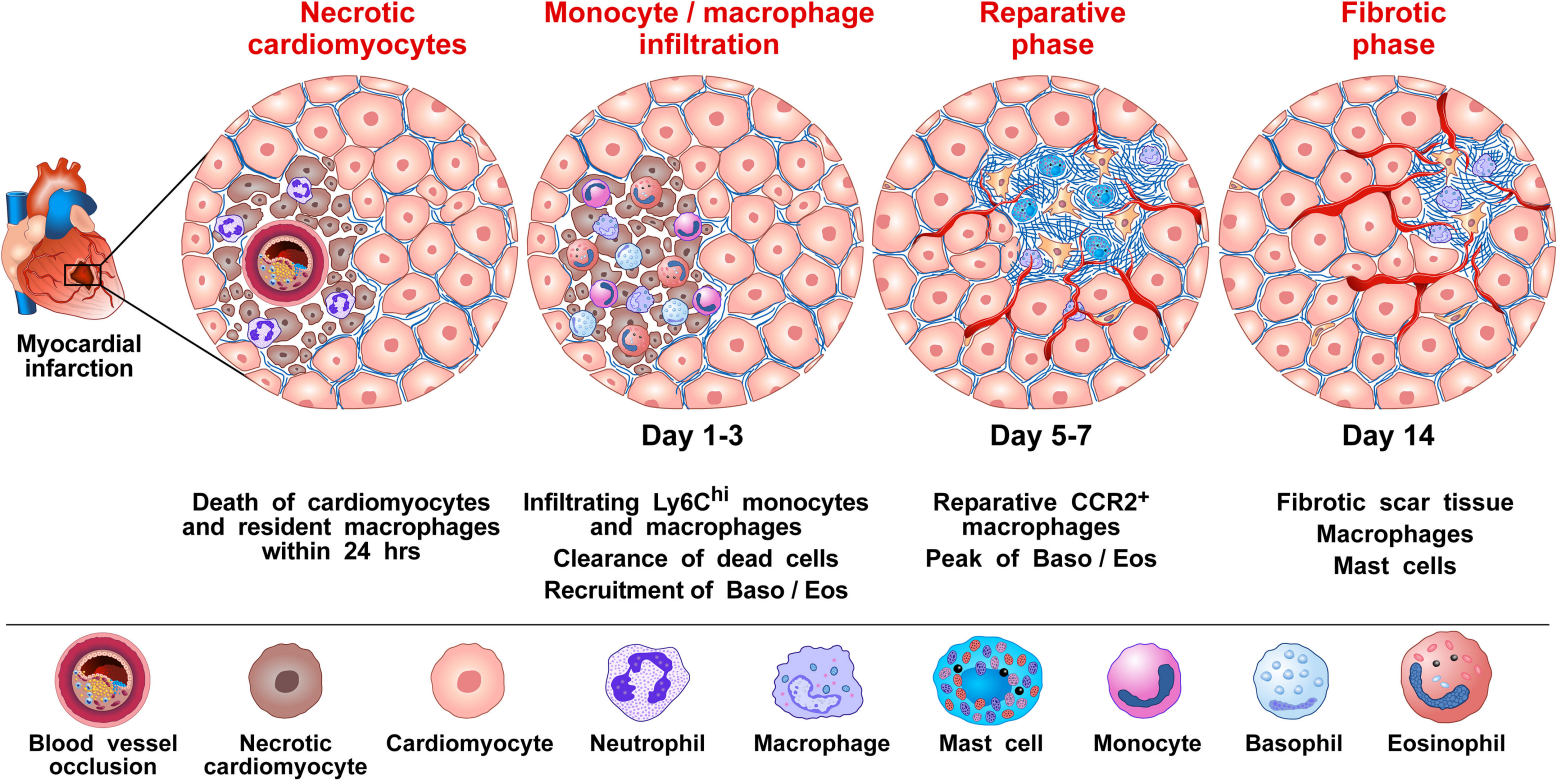
Proposed mechanism by which basophils influence the inflammatory response to promote wound healing and tissue repair following myocardial infarction (MI). MI is caused by the rupture of an atherosclerotic plaque causing the occlusion of a coronary artery, which then results in cardiac tissue damage due to ischemia (90). It has been shown in mice that several immune cells [e.g., monocytes/macrophages, neutrophils, dendritic cells (DCs), B and T cells, and natural killer (NK) cells, basophils and eosinophils infiltrate the heart after experimental MI (70, 97, 110). For basophils, this infiltration into the heart is evident 3 days following MI and peaks 7 days after the MI event (70). Monocytes/macrophages represent the most prevalent immune cells after MI. Cardiac resident macrophages contribute to the initial neutrophil infiltration into the ischemic area (111). Resident macrophages are reduced in murine models 1 day post-infarction (112). Within 1-3 days infiltrating bone marrow- and spleen-derived Ly6C^hi^ monocytes are recruited into the injured cardiac tissue and differentiate to Ly6C^low^ macrophages facilitating clearance of necrotic cardiomyocytes. At approximately 5-7 days post MI, macrophages adopt a reparative phenotype, contributing to the resolution of inflammation and fibrotic tissue formation (70). By day 3, infiltrating basophils into the injured cardiac tissue release IL-4 and IL-13, which induce phenotypical and functional changes within macrophages expressing anti-inflammatory and tissue repair genes (70). Formation of neovessels in the healing infarct play an important role in repairing the infarcted myocardium (113). Basophils (114), macrophages (115–117), and cardiac mast cells (118, 119), are major sources of angiogenic factors. Collectively, results in mice models of MI indicate that basophils infiltrating infarcted heart promote resolution of cardiac inflammation and scar formation.

Sicklinger et al. also studied the inflammatory response after MI in Mcpt8-Cre-transgenic (Baso-KO) mice constitutively deficient in basophils (57). In this model, the infarct size did not differ between Baso-KO compared to WT mice. However, 28 days after inducing the MI, the basophil-deficient mice developed cardiac dysfunction and increased heart weight compared to their WT littermates. Finally, Baso-KO mice showed increased scar thinning compared to controls. MI in genetic basophil ablation mice was associated with an altered cellular inflammatory response in infarcted hearts. Four days after MI, there was a change in the composition of monocyte subpopulations in the infarcted myocardium of the basophil-depleted mice, namely a shift from reparative Ly6C^lo^ macrophages toward inflammatory Ly6C^hi^ monocytes. This proinflammatory response could be reversed by the adoptive transfer of basophils into the basophil-deficient mice. The absence of basophils was associated with lower concentrations of cardiac IL-4 and IL-13, two cytokines typically released by mouse (9, 57, 102–105) and human basophils (9, 16, 36–38, 106–108). The authors concluded that the IL-4/IL-13 secreted by basophils infiltrating these lesions is critical in the transition from inflammatory monocytes to reparative macrophages (81, 109) **Figure 1** illustrates the proposed mechanisms by which basophils influence the inflammatory response following myocardial infarction.

The authors also evaluated the cytokines produced in the heart 3 days after the MI event, both in the Baso-KO and WT mice. Among the cytokines commonly reported to be produced by mouse basophils (IL-4, IL-13, IL-6, TNF-α), there was a reduction only of IL-4 in the injured heart tissue of the basophil-deficient mice. Mice-deficient in IL-4/IL-13 showed a higher proportion of inflammatory Ly6C^hi^ monocytes and worsened cardiac function following MI. In contrast, the increased release of IL-4 by basophils following the administration of the glycoprotein IPSE/α-1 (a known stimulus of these cytokines from basophils) resulted in enhanced post-MI healing. The authors concluded that myocardial basophils are activated to produce IL-4 following MI and that this response is critical in healing the damaged myocardium (70). What currently remains unknown, however, is the exact mode of stimulation in the myocardium responsible for inducing basophils to produce IL-4.

These experimental results were supported by observations that human subjects presented with decreased blood basophil numbers within the first week following an MI event, and that this basopenia associated with an increased scar size, as measured by late gadolinium enhancement cardiac MRI after one year of follow-up (70). Importantly, this correlation persisted after the adjustment of possible confounders (e.g., initial infarct size, systemic inflammation, cardiovascular risk factors). The authors suggested that basophils may also influence cardiac remodeling after MI in humans.

These studies, emphasizing the protective role of basophils following MI, might have translational relevance. For example, a growing number of allergic patients (e.g., asthma, atopic dermatitis) are being treated with biologics that block the IL-4/IL-13 axis (e.g., dupilumab, an anti-IL-4Rα mAb) (82, 120). Thus, the possible protective role of basophil-derived IL-4/IL-13 in MI should stimulate further mechanistic studies to investigate possible links between these therapies and whether they might impact myocardial healing following MI.

## 3 Basophils in kidney fibrosis

Chronic kidney disease (CKD) is a final manifestation of renal fibrosis and its incidence is increasing (121). Various inflammatory stimuli, including chronic infections, tissue injury, autoimmune disorders, chemical insults, and radiation result in kidney fibrosis (117, 122). Chronic low-grade inflammation is a crucial promoter of fibrosis (117, 123), but immune pathways orchestrating kidney fibrosis are largely unknown. Doke and collaborators investigated the interactions between altered renal tubules and basophils in a mouse model of kidney fibrosis by employing single-cell RNA-seq analysis (71). In this model of CKD, mice experienced either a sham operation or underwent unilateral ureter obstruction (UUO) surgery. Injured tubular cells (PTs) expressed several cytokines and chemokines known to induce the recruitment of basophils and other immune cells. PTs also released platelet-derived growth factor B (PDGFB), which upon binding to its receptor (PDGFBR) on fibroblasts induces these cells to release TGF-β. CXCL1, secreted by profibrotic tubules, recruited CXCR2^+^ basophils. The density of basophils (FcϵRI^+^CD200R3^+^CD49b^+^ cells) was markedly increased in UUO kidneys compared to sham operation. Using antibody-mediated and genetic approaches to delete basophils, the authors explored the role of these cells in this model. In the latter model, injection of diphtheria toxin (DT) into Mcpt8^Cre^/DTR mice induced depletion of basophils in the kidney and mitigated fibrosis in UUO kidney. Single-cell analysis and *in situ* hybridization demonstrated overexpression of *Il6* by basophils in UUO kidneys, indicating that mouse basophils are a source of this cytokine in UUO kidneys. In the other model, basophil depletion was mediated by MAR-1 administration into WT mice, followed by UUO surgery and kidney examination 7 days later. MAR-1-treated mice showed a reduction of the fibrosis markers induced by the UUO surgery. These results from two complementary models of basophil depletion highlight the importance of these cells in the development of experimental kidney fibrosis.

There is evidence that T_H_17 cells contribute to renal fibrosis (124). For example, basophils were shown to directly interact with T_H_17 cells and macrophages (104, 125). Both T_H_17 cell number and IL-17A expression were increased in UUO, but they were lower in UUO kidneys of basophil-depleted mice. Single-cell RNA-seq analysis indicated a shift toward T_H_17 cells in fibrosis. Basophil-derived IL-6 contributed to enhanced T_H_17 cell differentiation from CD4^+^ T cells in UUO kidney (126). Moreover, the expression of *Il17a* and *Tgfb1* were higher in UUO kidneys and were lower in UUO kidneys of basophil-depleted mice. Mice treated with an anti-IL-6R antibody were partially protected from renal fibrosis.

**Figure 2:**
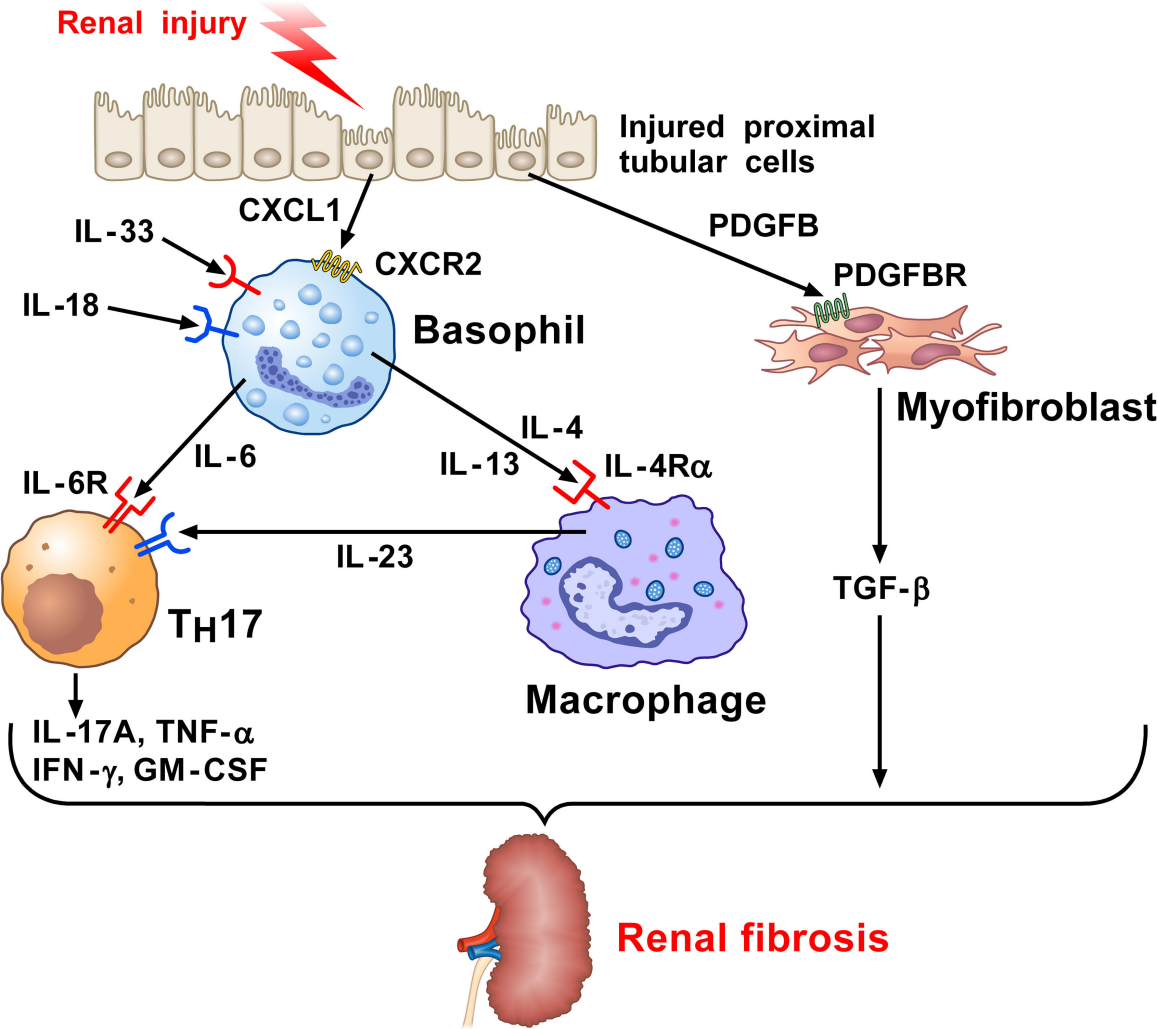
Kidney and wild-type mice subjected to unilateral ureter obstruction (UUO) surgery revealed the presence of neutrophils, monocytes/macrophages, dendritic cells (DCs), and basophils (71). Injured proximal tubular cells (PTs) in UUO kidney express *Il34*, *Cxcl10*, and the key profibrotic factor (71), platelet derived growth factor subunit B (PDGFB). PDGFB released by injured tubular activates the PDGFB receptor (PDGFBR) on fibroblasts to release TGF-β. Profibrotic PT cells participate in the recruitment of myeloid and lymphoid cells and the local fibroblast activation. CXCL1 released from PT cells induces the recruitment of basophils through the engagement of CXCR2. Basophils in UUO kidney can be activated by IL-33 and IL-18 released from the stroma to secrete IL-6. This cytokine favors T_H_17 differentiation from CD4^+^ T cells in UUO kidneys. IL-17A and TGF-β released from T_H_17 cells contribute to renal fibrosis. IL-4 and IL-13 released from activated basophils can contribute to macrophage activation (127–130). PDGF released from injured PT cells activates the PDGFR on myofibroblasts causing the release of TGF-β. Macrophages are also a major source of IL-6. Collectively, these findings indicate that basophils and their mediators contribute to kidney fibrosis.

To evaluate the relevance of the above experimental findings to human kidney fibrosis, Doke and collaborators examined human kidneys, comparing those from healthy controls and CKD subjects using single-cell RNA-seq (71). They found that basophil numbers were increased in the kidney of patients with CKD, compared to healthy controls. Moreover, a correlation between renal fibrosis and basophil density was evident in the kidneys of CKD patients. There was also a positive correlation between *IL6* expression and the severity of renal fibrosis, which further showed a negative correlation between *IL6* and kidney function. Moreover, renal *IL6* correlated with CKD severity. Collectively, the above results reveal several correlations between both basophil density and their function and renal fibrosis. **Figure 2** schematically illustrates the contribution of basophil-derived cytokines and T_H_17 as downstream mediators in kidney fibrosis.

## 4 Basophils in cancer

There is mounting evidence showing that basophils are an important component within the tumor microenvironment (TME) of several human (72, 88, 131, 132) and mouse experimental cancers (72, 73, 132, 133). Moreover, these studies indicate that basophils may play an active role in the onset and development of both solid and hematologic tumors (74, 86, 134). The results from these studies reveal that basophils can have both pro-tumor and antitumor effects depending on the context and type of tumor.

In particular, immune profiling studies show that basophils constitute a portion, albeit small, of the immune landscape in human non-small cell lung cancer (NSCLC) tumors (131) and in the immune infiltrate seen in the early stage of lung adenocarcinoma (132). Several studies additionally show that mouse and human basophils support the development and expansion of M2-like monocytes/macrophages (127–130), which are often prevalent in the TME favoring tumorigenesis. An *in vivo* study in mice points to the importance of IL-4/IL-13, promoting carcinogenesis by reducing Th1-like immunity (72). Likewise, basophils are known to secrete vascular endothelial growth factor-A (VEGF-A) (114) and cysteinyl leukotriene C_4_ (LTC_4_) (18, 19) with the latter more recently implicated in tumorigenesis and metastasis formation (135). In particular, both tumor growth and metastases were reduced in mice deficient in the cysteinyl leukotriene 2 receptor (CysLT_2_R). Moreover, administration of a CysLT_2_R antagonist reduced tumor growth and metastases in WT mice (135).

In exploring the immune cells involved in human pancreatic cancer (PC), *IL4-*expressing basophils were identified in the tumor-draining lymph nodes (TDLNs). Moreover, their presence was a negative prognostic marker of patient survival (72). To further investigate the underlying mechanisms of this association, the *Mcpt8* ^Cre^ basophil deficient mouse strain (57) and WT mice were implanted with PC cells. Strikingly, 80% of the WT mice developed PC-like cancer, but this was not observed in the basophil-deficient mice (72). The authors reported that TSLP released from basophils and cancer-associated fibroblasts (CAFs) within TDLNs activated CD4^+^ T cells to produce IL-3. CCL7, derived from DCs and monocytes, promoted basophil recruitment into TDLNs. IL-3-activated basophils exerted a pro-tumorigenic role by secreting IL-4, which induced the polarization of Th2 and M2 cells. Thus, these results not only confirmed/supported the notion that basophil-derived IL-4/IL-13 promote Th2 and M2-like cells, but also demonstrated that these cells actively participate in promoting PC.

With the concept that various basophil-derived products (e.g., IL-4, IL-13, VEGF-A, LTC_4_) promote tumorigenesis, an equally important issue pertains to the stimuli mediating their release. Schroeder and colleagues have shown that human basophils release copious amounts of histamine, IL-4 and IL-13 when co-cultured with the human lung adenocarcinoma cell line A549 (16). These responses were dependent on basophils expressing IgE, since removal/depletion of this immunoglobulin prevented basophil activation. Since pharmacologic inhibitors of FcϵRI signaling also suppressed these responses, it seemed clear that basophils were being activated *via* IgE/FcϵRI crosslinking to secrete these cytokines. Importantly, direct contact between basophils and A549 was necessary and occurred even if the adenocarcinoma cells were fixed with paraformaldehyde prior to co-culture. In a follow-up study, the IgE-binding lectin, galectin-3 (Gal-3) expressed on the A549 cells, proved crucial for basophil activation in these co-cultures, as A549 clones lacking Gal-3 failed to activate basophils (136). Gal-3 is widely implicated in various cancers and is a marker of chronic inflammation (137). These findings reveal a potentially new mechanism by which Gal-3 expressed by human lung adenocarcinoma cells can activate basophils to release cytokines and pro-inflammatory mediators that promote tumorigenesis. Additional investigations are required to fully understand all aspects of this mechanism and how it might be targeted for therapeutic intervention.

By utilizing a model whereby the skin of mice were topically exposed to the proinflammatory 12-0-tetradecanoylphorbol-13-acetate (TPA), Hayes et al. showed that serum IgE increased in these animals, which was accompanied by increased numbers of IgE-bearing basophils that promoted skin tumorigenesis (73). In a similar model of epithelial carcinogenesis involving the use of [7,12-dimyethylbenz(a)anthracene (DMBA) and subsequent exposure to TPA], mice lacking IgE (*lgh7^-/-^*) developed less tumors compared to WT mice. The influx of basophils into skin was promoted by CXCR4, TSLP and IL-3. IgE-signaling played a key role in basophil activation and infiltrating tissue basophils expressed *Cxcr2, Cxcr4*, and *Ptgdr2* (CRTH2, the PGD_2_ receptor). Tumor development was markedly reduced when conducting the same experiment in *Mcpt8* ^Cre/+^ mice, which were made deficient in basophils but retained normal mast cell numbers (57). Collectively, these *in vivo* results further indicate that FcεRI-signaling in basophils promotes inflammation-driven epithelial hyperplasia and tumor growth. While the role of galectin-3 in this tumorigenesis was not investigated, it seems worthy of future investigation, as mechanisms of this response are further elucidated.

In contrast to the belief that basophils contribute to tumorigenesis, association studies have shown evidence that higher expression of basophils (i.e., CD123^+^, CCR3^+^, FcεRI^+^) in tumors correlated with better overall survival (88). In particular, increased basophil numbers are associated with beneficial outcomes in several cancers, including sarcoma, lung, and breast. While several additional markers (e.g., CD63, CD203c) indicated that these tumor-associated basophils were, indeed, activated, relevant mediators commonly released by these cells (histamine, LTC_4_, IL-4, IL-13) were not investigated. Thus, the exact contribution of basophils in the increased survival rates remains challenging to interpret at this time. Likewise, the same group has reported evidence that the *in vitro* responses of peripheral blood basophils from cancer patients can predict survival rates. While such correlations are intriguing, the exact mechanisms by which basophils contribute to increased survival rates is an area requiring further elucidation.

In agreement with the concept that basophils mediate a beneficial role in cancer, evidence from a mouse melanoma model showed that basophils released CCL3 and CCL4, which induced CD8^+^ T cell recruitment and promoted tumor rejection (75). MAR-1 administration in these melanoma-bearing mice depleted basophils and prevented melanoma rejection. However, it is important to note that basophil depletion using the MAR-1 is also reported to deplete/activate other immune cells expressing FcϵRI, including mast cells, monocytes and DCs (138, 139). Whether these cells were also depleted and possibly involved in tumoricidal activity remains unclear.

IL-33 has been shown to promote tumoricidal activity mediated by eosinophils (140, 141), possibly by upregulating granzyme B (142). As noted, this cytokine also activates both human and mouse basophils (9, 36, 38, 143–145). Hence, IL-33-activated basophils co-cultured with B16.F10 melanoma cells were shown to inhibit tumor growth compared to melanoma cells co-cultured with un-stimulated basophils (142).

**Figure 3:**
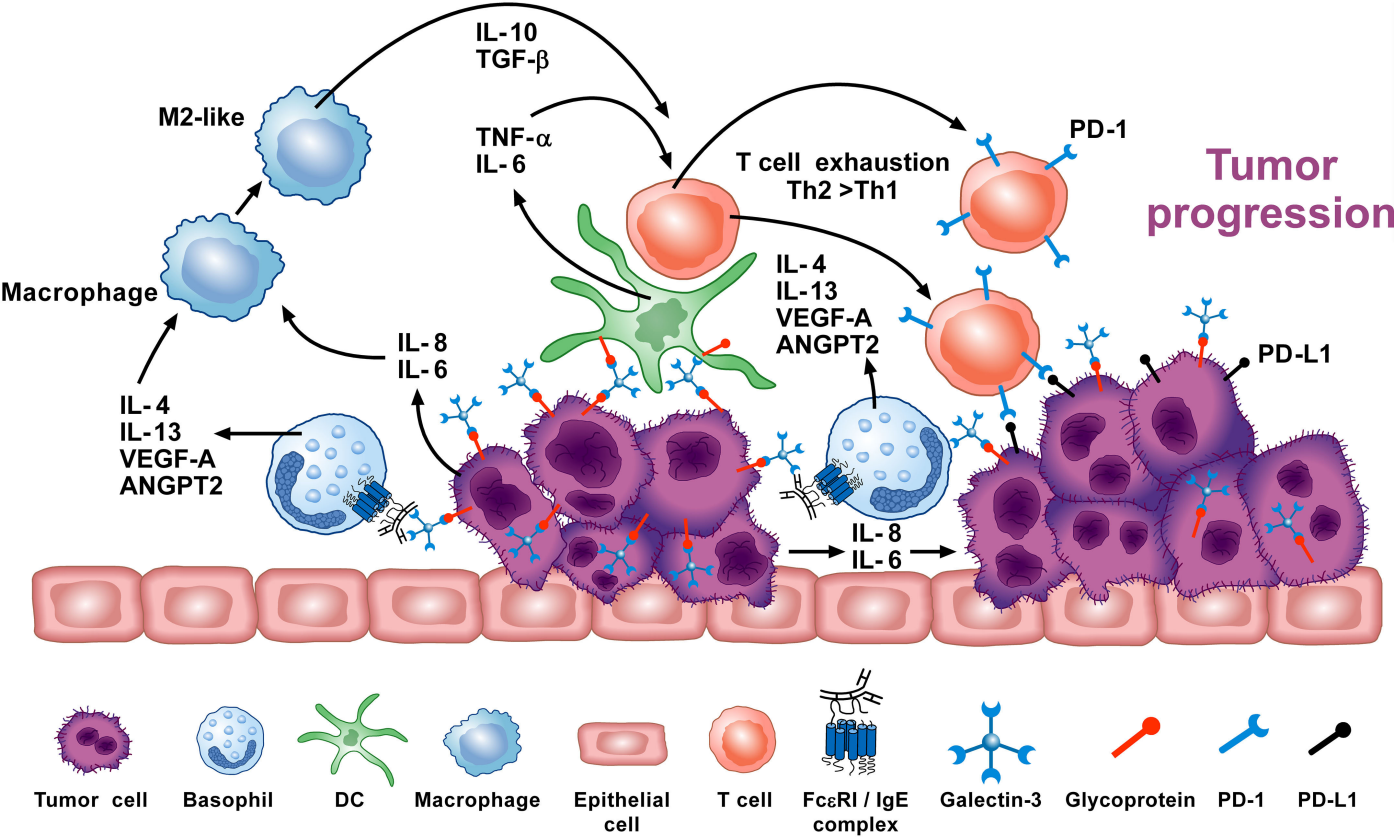
Basophils can promote tumor progression through different mechanisms. Galectin-3 (Gal-3) is a lectin expressed by several cancer cells (137), including the A549 adenocarcinoma cell line (EC-Gal-3). Gal-3 activates human basophils to release IL-4 and IL-13 (16, 136), which are widely known to promote M2-like macrophages, the major players in the TME (127–130). IL-4^+^ basophils have been found in the TME of human and experimental pancreatic cancer (72). Human and mouse basophils also secrete VEGF-A and angiopoietin 2 (ANGPT2) that can promote tumor angiogenesis (114, 146–148). Basophils can promote IL-6 and IL-8 release from epithelial cell lines through a mechanism requiring cell-to-cell contact (149) (JTS, unpublished). Tumor cell-derived IL-6/IL-8 play a critical role in metastasis formation (150). Dendritic cells and monocytes activated by EC-Gal-3 release TNF-α and IL-6 *in vitro* (151). These cytokines, combined with M2 cell-derived IL-10 and TGF-β induce T-cell exhaustion by up-regulating checkpoint inhibitors (i.e., PD-1), which interact with tumor cell-associated PD-L1 to decrease cytotoxic T cell activity (152, 153). These results suggest that basophils can promote tumorigenesis in certain experimental and clinical conditions. Adapted from Poto et al. (74).

**Figure 4:**
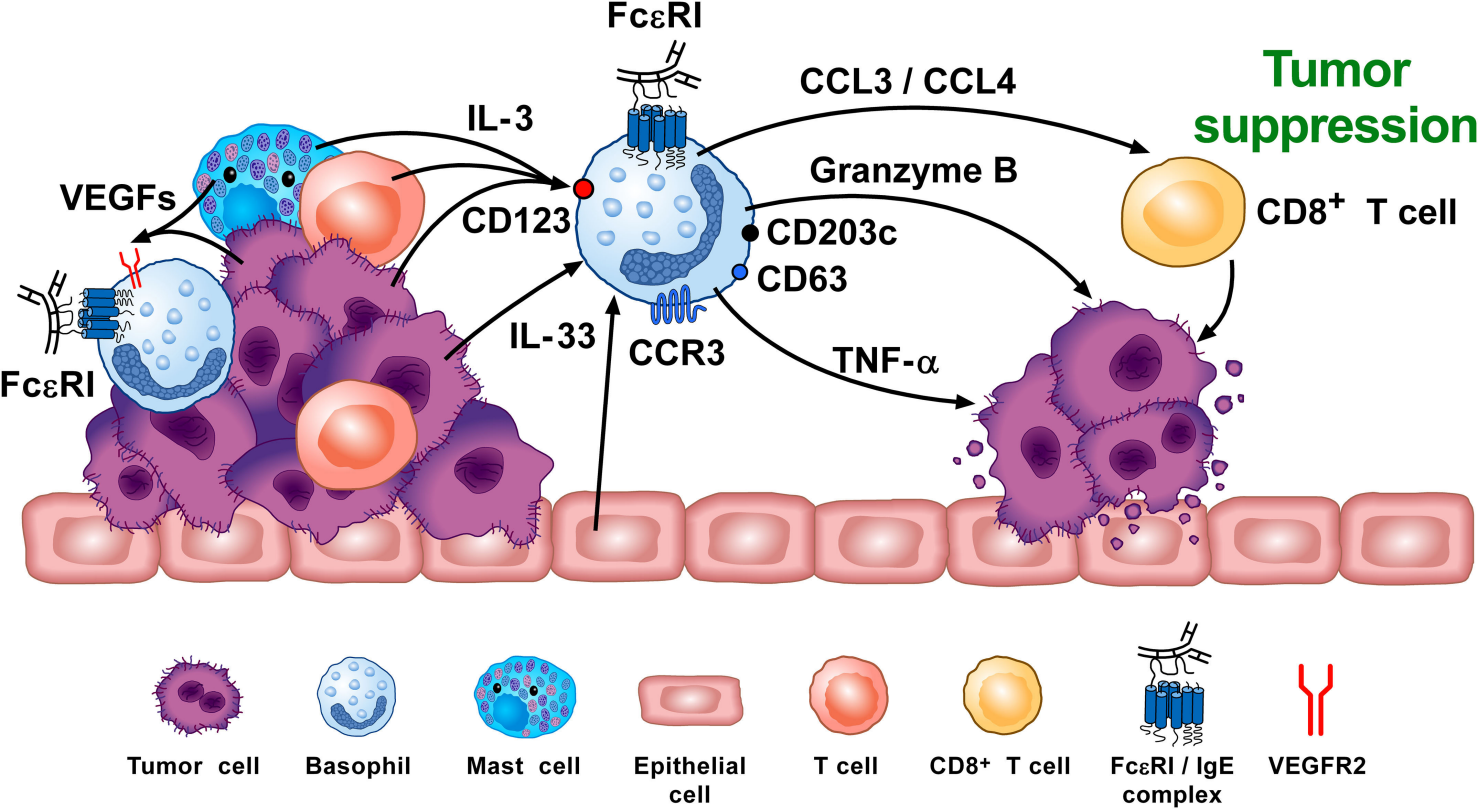
Basophils can promote tumor suppression through different mechanisms. Vascular endothelial growth factors (VEGFs) released by tumor and immune cells in the TME (e.g., macrophages, mast cells) (155–159) induce basophil recruitment via the activation of VEGFR2 on these cells (155). IL-3, released from intratumoral lymphocytes, mast cells and tumor cells (10, 160, 161), is the major growth, differentiation, priming and activating factor for both human and mouse basophils via the activation of the IL-3 receptor (IL-3Rα/CD123) (8–10). Intratumoral basophils secrete CCL3 and CCL4 which favor CD8^+^ T cell infiltration in TME, favoring melanoma rejection in mice (75). IL-33 produced by epithelial and tumor cells, plays a critical role in tumorigenesis (162) by upregulating granzyme B mRNA and the surface expression of CD63 in basophils. Mouse basophils activated by IL-33 cause melanoma cell death *in vitro* (142). Mouse (104, 163) and, in certain conditions, human basophils (164, 165) release TNF-α and granzyme B (142, 166), which exerts cytotoxic activity on cancer cells (102, 167). Tumor resident basophils overexpressing CD123, CCR3, CD63, CD203c mRNAs are associated with improved outcome in ovarian cancer (88, 154). These findings indicate that, under specific experimental and clinical circumstances, basophils can play an anti-tumorigenic role. Adapted from Poto et al. (74).

Overall, there are several studies indicating that basophils promote tumorigenesis (72, 74). In this instance, the tumor cells cause basophils to release cytokines/chemokines that may facilitate the development of protumorigenic TME (**Figure 3**). Interestingly, many of the same TME elements involved in this activity (e.g., IL-4, IL-13, galectin-3, VEGF-A, M2 and Th2 cells) are also implicated in promoting wound healing. Conversely, in certain tumors (e.g., melanoma), basophils mediate anti-tumor effects (75, 88, 154) (**Figure 4**). The mechanisms underlying the protective effects of basophils remain largely unknown. It has been suggested that certain mediators (e.g., TNF-α and granzyme B) released by basophils exert tumoricidal effect. In addition, other molecules (e.g., CCL3 and CCL4) can favor the recruitment of cytotoxic CD8^+^ T cells (74). Collectively, these findings highlight some apparently conflicting results regarding the role that basophils potentially exert in different models of tumorigenesis, and thus warrant further investigation.

## 5 Basophils in autoimmune disorders

### 5.1 Systemic lupus erythematosus

With the discovery of IgE (168, 169), immunologists focused their attention on understanding its relevance for allergic disorders and host defense against parasitic infestations (2, 64, 81, 170). However, circulating IgE autoantibodies in rheumatoid arthritis and SLE patients had been reported as early as the late 70’s (171). While these early studies were conducted mostly using small cohorts of patients, they did confound the thought at the time that atopy was generally limited to patients suffering from allergic disease and/or parasitic infestations.

Systemic lupus erythematosus (SLE) is an autoimmune disorder associated with circulating self-reactive antibodies (172) (i.e., IgG anti-double-stranded DNA: anti-dsDNA). Several studies reported increased serum IgE in SLE, which correlated with severe disease manifestations (76, 173–175). A portion of the circulating IgE in these SLE patients was determined to be self-reactive, binding to nucleic acids, as was often the case for most IgG autoantibodies (176). In fact, several studies identified IgE against at least one autoantigen in SLE patients (171, 173, 177–182). Importantly, IgE anti-dsDNA antibodies are associated with disease activity and hypocomplementemia (177). Moreover, the levels of IgE anti-dsDNA proved to be an independent risk factor for SLE activity, even after excluding the levels of IgG anti-dsDNA (178). One study reported that IgE anti-dsDNA antibodies are found in ~ 70% of lupus patients, and are possibly linked to kidney damage (178). In a Franco-American cohort, IgE anti-dsDNA antibodies did associate with lupus nephritis, whereas IgE against other nucleic acid–containing autoantigens (Sm, SS-A/Ro, and SS-B/La) did not associate with disease (177). These findings suggested that IgE autoantibodies could play a role in the pathophysiologic mechanisms of lupus nephritis. The French-American collaborative study identified IgE autoantibodies against three new autoantigens: APEX nuclease 1, N-methylpurine DNA glycosylase and CAP-Gly domain-containing linker protein family member 4. These autoantigens specifically elicited IgE autoantibodies but not IgG autoantibodies (177). Collectively, these results indicate that IgE autoantibodies are prevalent in lupus nephritis patients and are associated with disease activity. Likewise, these findings provided the impetus for treating SLE patients in a randomized clinical trial using anti-IgE mAb (omalizumab) (NCT01716312).

**Figure 5:**
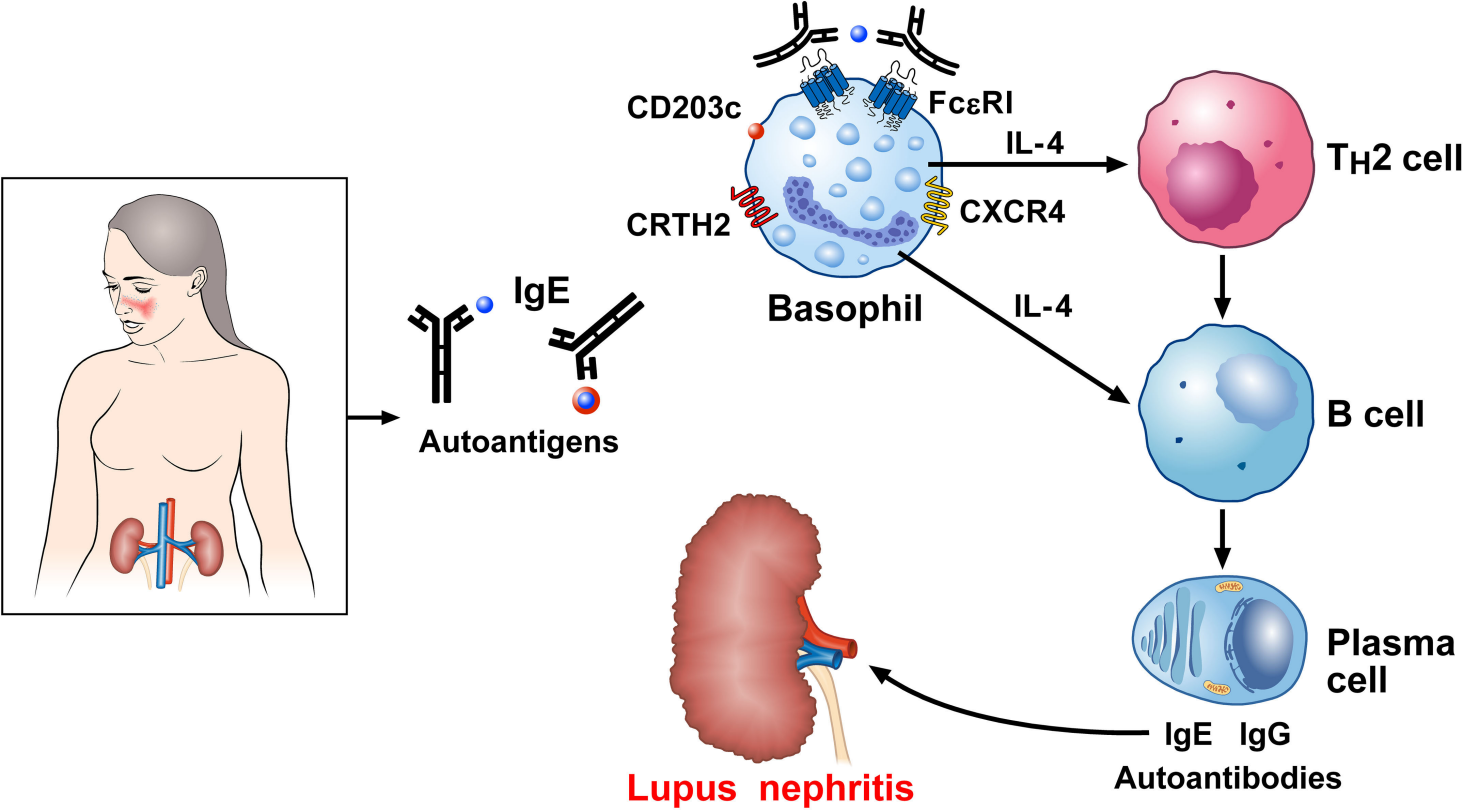
Proposed mechanism linking IgE basophils to autoimmunity in systemic lupus erythematosus (SLE). Serum IgE levels are increased in SLE and correlate with severe disease manifestations (76, 173–175). IgE against several autoantigens have been reported in SLE (171, 173, 177–182). Basophils from SLE patients show an activated phenotype in overexpressing CD203c (76), the prostaglandin D_2_ (PGD_2_) receptor [chemoattractant receptor-homologous molecule (CRTH2) expressed on Th2 cells], and CXCR4, the receptor for CXCL12 (185). Once recruited to the secondary lymphoid organs, activated basophils release IL-4, which drives B cell isotype switching toward IgE and autoreactive IgE (177, 181). Dendritic cells (DCs) in lymph nodes also act on B cells, triggering their differentiation into plasma cells and potentiating the formation of self-reactive autoantibodies (186). IgE immune complexes contribute to basophil activation. Deposits of IgG and IgE autoantibodies in the kidney play a major role in lupus nephritis.

Charles et al. first demonstrated mechanistic evidence that basophils are implicated in the pathobiology of lupus nephritis by using a spontaneous murine model of SLE (Lyn^-/-^ mice) (76). This observation was subsequently confirmed using a model of pristane-induced lupus-like nephritis (183) as well as in a cohort of SLE patients (181). Basophils from SLE patients express significantly higher levels of the basophil activation marker, CD203c, compared to healthy controls (76). It was also found that the basophil density in both lymph nodes and spleen of SLE patients was higher than controls. Basophil-derived IL-4 reportedly induced B cell class switching toward IgE, and the autoreactive IgE produced was determined to be a relevant inducer of lupus (177, 178, 181, 184). Basophils from human patients with SLE and from two different lupus-like mouse models, overexpress both PGD_2_ receptors (PTGDR-1 and PTGDR-2) and CXCR4, the receptor for CXCL12 (185). Basophils seemingly contribute to SLE pathobiology by migrating to secondary lymphoid organs in a prostaglandin D_2_ (PGD_2_)- and CXCL12-dependent manner (185). These basophils can then support plasma cell functions by amplifying the production of autoantibodies and circulating immune complexes (76, 183, 185). **Figure 5** schematically illustrates the mechanisms presumably linking IgE and basophils to SLE.

### 5.2 Rheumatoid arthritis

Rheumatoid arthritis (RA) is a systemic autoimmune disease primarily involving inflammation of the joints (187). On a genetic background (i.e., HLA-DR4 found in ~ 70% of RA patients compared to 30% of controls), post-translational citrullination of several self-proteins generates altered self-antigens that activate CD4^+^ T cell responses in RA patients. Citrullination occurs *via* the conversion of arginine into citrulline by peptidyl arginine deiminases (PADs). Anti-citrullinated protein antibodies (ACPAs) are specific and predictive for RA and are implicated in the pathogenesis of RA (187).

IgE antibodies against citrullinated fibrinogen were detected in the serum of ~ 60% of ACPA^+^ RA patients (188). These authors reported that basophils from ACPA^+^ RA patients can be activated by citrullinated protein, whereas basophils from healthy controls were not activated. Serum from IgE-ACPA^+^ RA patients passively sensitized human FcϵRI^+^ expressing rat basophil cells (RBL) for activation by citrullinated proteins. These finding indicate that basophils from IgE-ACPA^+^ RA patients can be activated by citrullinated antigens. The results of this original study deserve to be extended using citrullinated proteins specific for RA patients.

### 5.3 Autoimmune encephalomyelitis

Experimental autoimmune encephalomyelitis (EAE) is an animal model widely used to investigate the mechanisms underlying multiple sclerosis (MS) (189). EAE differs from MS in needing to be induced rather than occurring spontaneously, although recent transgenic mouse models have indicated spontaneous development of EAE (189, 190). However, inoculation with central nervous system antigens and adjuvant or passive transfer of lymphocytes reactive with these antigens are often employed to induce EAE in many animal strains (189).

Yuk and collaborators have investigated the mechanisms by which basophils can contribute to T_H_17 differentiation and EAE pathogenesis (126). For example, IL-17 is highly expressed in MS lesions (191) and T_H_17 cells mediate blood-brain barrier disruption and the expression of IL-17 and IL-22 (192). T_H_17 differentiation requires IL-6 and TGF-β (193), yet whether basophils promote T_H_17 induction in EAE had remained unknown. To address this possibility, Yuk and coworkers demonstrated that IgE cross-linking, or the use of cholera toxin (CT), induced the release of IL-6 and IL-4 from bone marrow-derived basophils (126). Moreover, they found that basophils mediate T_H_17 differentiation through IL-6 secretion. The authors also examined whether basophils contribute to T_H_17 polarization *in vivo*. WT and IL-6-deficient mice were challenged with CT plus antigen. IL-17A producing CD4^+^ T cells were reduced in IL-6 deficient animals, suggesting that IL-6 is critical for the antigen-induced T_H_17 response. The role of basophils was also examined in basophil-deficient mice. The authors found that basophil-derived IL-6 cooperates with DCs to promote the differentiation of CD4 T cells into T_H_17 cells. T_H_17 responses were reduced in the absence of basophils or IL-6. Collectively, these findings suggest that basophil-derived mediators (e.g., IL-6) are involved in T_H_17 cell differentiation, allowing T_H_17 cells to migrate to the site of inflammation mediating pathogenic functions in EAE. These studies identify basophils and their mediators as candidates for investigating pathogenic mechanisms in MS patients. It should be noted that EAE pathology is not driven exclusively by T_H_17 and IL-17; other cells (e.g., CD8^+^, T cells, γδ T cells) and cytokines may also be involved (194).

### 5.4 Mixed connective tissue disease

Mixed connective tissue disease (MCTD) is a rare systemic autoimmune disease (incidence ~ 2 per 100,000 adults) affecting mainly women (~ 90%) (195). Its clinical manifestations often overlap with other connective tissue disorders, including SLE, systemic sclerosis, or myositis (196). The defining immunological feature of MCTD is the presence of autoantibodies recognizing the 70-kDa subunit of the U1 small nuclear ribonucleoprotein (U1-snRNP 70k) in the absence of IgG against dsDNA or to Sm, two SLE hallmarks (197). The pathophysiology underlying MCTD remains poorly understood, but posttranslational modifications of U1-snRNP are known to generate neoepitopes that may contribute to the disease (198). These neoepitopes can result in T cells recognizing U1-snRNP, which ultimately lead to the induction and proliferation of autoreactive B cells synthesizing autoantibodies (199). Immune complexes made of anti-U1snRNP antibodies and their antigen can activate endothelium and immune cell *via* a variety of receptors (e.g., Fc, complement, and Toll-like receptors, TLR), resulting in vascular disease and tissue injury (200–203). Pulmonary involvement characterizes more than 70% of MCTD patients (197). A mouse model has been described whereby mice immunized with human U1-snRNP develop a MCTD-like lung disorder (204).

Lamri and collaborators observed that basophils from patients with MCTD present an activated phenotype (77), sharing some features with basophils from SLE patients (i.e., overexpression of CD203c, CXCR4) (76, 185). In addition, basophils from MCTD expressed increased surface markers such as CCR3, yet unchanged expression levels of CD62L (77). A similar basophil phenotype was found in a MCTD-like mouse model in which activated basophils infiltrated in the lungs and lymph nodes. To study the contribution of basophils in the development of lung pathology in this model, basophils were depleted through the injection of DT in female Bcpt8^DTR^ mice. Basophil depletion reduced the cellular infiltrates (e.g., CD4^+^ T cells) in the lungs. The authors also examined the MCTD-like lung disease in IgE-deficient mice (*Igh7* ^-/-^). Similar to that seen with basophil depletion, IgE deficiency also protected mice from developing immune cell infiltration and lung fibrosis. These results indicate that basophils play a major effector role in inducing lung fibrosis *via* an IgE-dependent mechanism. The authors suggested that basophils, activated by the U1-snRNP antibodies complex, accumulate in the airways, where they release IL-4 contributing to lung fibrosis development. In this scenario, IgE-mediated basophil activation may play both immunoregulatory and effector roles in the development of MCTD lung disease. These mouse models identify basophils, and IgE as candidates for investigating pathogenic mechanisms in patients with MCTD.

## 6 Basophils in IgG4-related disease

IgG4-related disease (IgG4-RD) is a rare multi-organ disorder characterized by lympho-plasmacytic infiltration, fibrosis, and obliterative phlebitis (205, 206). This condition is characterized by IgG4^+^ plasma cell infiltration in different organs (e.g., biliary tree, pancreas, retroperitoneum, salivary and lacrimal glands, and lymph nodes) (207, 208). The disease was first described in 2003 in a cohort of seven patients with a diagnosis of autoimmune pancreatitis (AIP) associated with IgG4^+^ plasma cell infiltration (209). Although the pathogenic mechanisms underlying IgG4-RD remain elusive (206), an increased production of Th2 cytokines (IL-4, IL-5, IL-13) has been identified in IgG4-related cholangitis and pancreatitis (210). These cytokines favor IgE production and eosinophil recruitment. It has also been reported that in patients with IgG4-RD, there is an accumulation of T regulatory cells (Tregs) in the blood, along with evidence that these cells infiltrate affected tissues, showing overexpression of IL-10 and TGF-β (211, 212). TGF-β released from Tregs can stimulate fibroblasts to produce collagen. IL-10 produced by Tregs can also stimulate secretion of IgG4 from plasma cells. The involvement of IL-10 and TGF-β secreting basophils has been suggested in patients with IgG4-related submandibular gland disease (213). B cell activating factor (BAFF) and APRIL, in combination with IL-21, can promote the expansion of IgG4-committed B cells (214, 215).

Two studies performed by different investigators in Japan proposed a possible mechanism whereby basophils are stimulated *via* a TLR-dependent activation involving IgG4-RD (214, 216). When activated by TLR2/TLR4 agonists, basophils from healthy donors induced B cells to produce IgG4 and IgG1 (214). TLR4 activation of basophils induced the release of IL-13 and BAFF. Basophils from IgG4-RD patients, upon activation with TLR2 and TLR4 ligands, induced more IgG4 than IgG1 when co-cultured with B cells. The authors suggested that the activation of TLRs in basophils play a role in IgG4-RD development (214).

Another study examined the role of basophils from peripheral blood and pancreatic tissue in patients with autoimmune pancreatitis (AIP) (216). AIP is a manifestation of IgG4-RD (208). Basophil density in the pancreas of AIP patients was higher than in alcoholic pancreatitis (216). In some of these patients, peripheral blood and intrapancreatic basophils were TLR2 or TLR4 positive. The authors suggested that basophils activated by TLRs could play a role in AIP. At present, the possible involvement of basophils and their mediators in the pathogenesis of different localizations of IgG4-RD remains unknown.

## 7 Basophils in chronic obstructive pulmonary disease

Chronic obstructive pulmonary disease (COPD) is a primary cause of morbidity and mortality worldwide (217). COPD is characterized by chronic inflammation, progressive airflow limitation and emphysema. Relative to asthma, the cellular and molecular mechanisms of COPD remain ill defined (117). It also differs in being characterized by a non-reversible airway obstruction (82, 218).

Shibata and collaborators elegantly investigated the potential role of basophils and their mediators in an elastase-induced murine model of COPD (62). Intranasal elastase elicited the recruitment of monocytes to the lung, followed by differentiation into interstitial macrophages (IMs) rather than alveolar macrophages (AMs). Matrix metalloproteinase-12 (MMP-12) played a key role in developing elastase-induced emphysema and was mainly expressed by IMs. The expression of *Il4*, but not *Il10*, *Il13*, or *Tgfb* was upregulated in the lung after the instillation of elastase. Expression of *Il4* mRNA was detected mainly in basophils, which accumulated in the lung. The authors used two complementary methods to deplete basophils *in vivo*, namely: diphtheria toxin (DT) treatment of *Mcpt8* ^DTR^ mice and anti-CD200R3 antibody treatment of WT mice. Using these models, they demonstrated impaired emphysema formation in basophil-depleted mice. They suggested that basophil-derived IL-4 promoted the differentiation of infiltrating monocytes into MMP-12–producing IMs that caused the alveolar wall destruction and emphysema formation. The authors concluded that the basophil-derived IL-4/monocyte–derived IM/MMP-12 axis plays a role in emphysema development. They also proposed that this novel cellular and humoral axis may be a potential target for COPD treatment.

In other findings, both eosinophils and basophils have been detected in several lung compartments of COPD patients, particularly in very severe COPD (219). Eosinophilic infiltration was patchy, and mainly confined eotaxin signatures with CCL11^+^ fibroblasts and CCL24^+^ macrophages. Basophils were preferentially localized in lymphoid tissue. These studies identify basophils and perhaps eosinophils as candidates for future investigations on their role in the pathogenic mechanisms of COPD.

## 8 Basophils in COVID-19

The current COVID-19 pandemic is caused by the novel severe acute respiratory syndrome Coronavirus 2 (SARS-CoV-2) (220). A dysregulated innate immune response is a key driver of clinical complications culminating in COVID-19 (221, 222). High levels of several cytokines (e.g., IL-1, IL-6, TNF-α, CXCL8) are detected early after viral infection, and many of these mediators are associated with granulocyte activation (223). The recombinant S1 subunit of the SARS-CoV-2 Spike protein activated *in vitro* human peripheral blood monocytes to release several cytokines (e.g., IL-6, IL-1β, TNF-α) and chemokines (e.g., CXCL10/IP-10, CCL3/MIP-1α, CCL4/MIP-1β) linked to COVID-19 (224). In this study, the S1 subunit did not induce any of these cytokines/chemokines from highly purified basophils (224). Another study reported that live SARS-CoV-2 virus induced IL-4 and IL-13 release *in vitro* from unprimed and IL-3-primed basophils (225). Although basophils have been implicated in the host response to other viruses (119, 226–229), the *in vivo* significance of basophil-derived cytokines/chemokines in the pathogenesis of COVID-19 remains unclear.

A detailed analysis at the single-cell resolution of granulocyte diversity in peripheral blood of COVID-19 patients demonstrated an increased level of both mature and immature neutrophils (230). By contrast, decreased basophils and eosinophils are often associated with severe COVID-19 (230, 231). Moreover, the emergence of PD-L1 expression on peripheral blood basophils (as defined as CD11b^+^SS^low^CrTH2^+^ cells) has been associated with COVID-19 severity (232). It should be pointed out that *in vitro* incubation of live SARS-CoV-2 with basophils purified from normal donors did not induce the expression of PD-L1 (225), whereas INF-γ increased PD-L1 expression on IL-3-primed basophils (233). High basophil counts are associated with a lower risk of developing severe COVID-19 (234). Collectively, these interesting results potentially implicate that basophils and/or their mediators play a protective role in COVID-19.

## 9 Basophils in inflammatory bowel diseases

**Figure 6:**
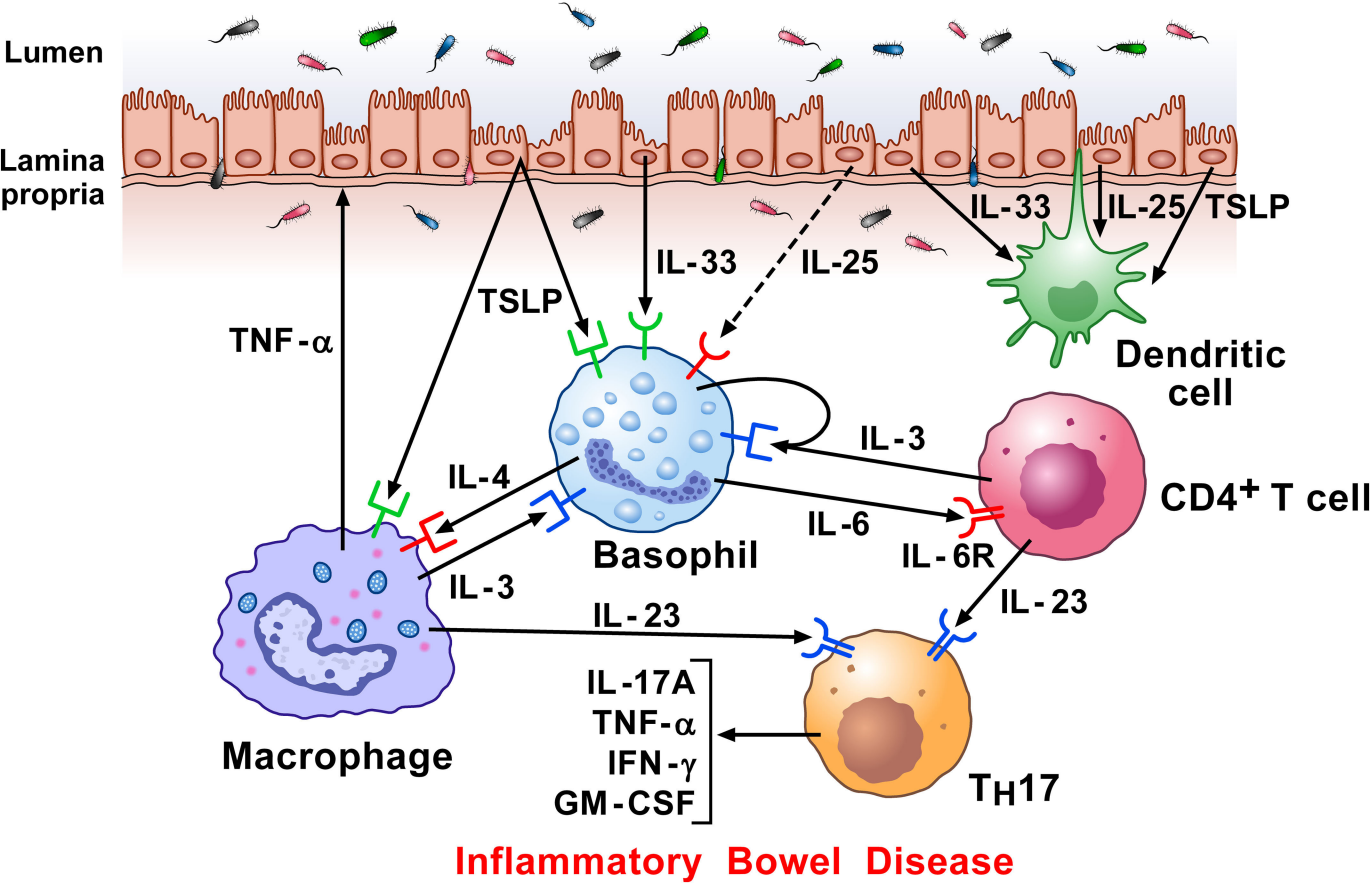
Hypothetical mechanisms by which dysregulated epithelial cells and inflammatory signaling by lamina propria immune cells in response to microbiota, contribute to inflammatory bowel disease (IBD) pathogenesis. Intestinal epithelial cells separate the lamina propria and deeper tissues from the luminal environment containing the intestinal microbiota (239). Increased intestinal permeability can potentiate immune-mediated systemic and intestinal inflammation in IBD (240). Damaged epithelial cells release alarmins (IL-33, TSLP, and IL-25) (115, 123, 241), which then regulate underlying immune cells (242), including basophils (9), macrophages (157), and DCs (243). Macrophages can damage epithelial cells directly by TNF-α secretion. Basophils accumulate in inflamed IBD compared to non-inflamed mucosa and to colon of healthy controls (125). Activated T cells infiltrate inflamed colons and release IL-3 which can contribute to the attraction and/or survival of basophils locally (238). Specific components of gut microbiota induce the emergence of intestinal T_H_17 cells. Basophils may also promote T_H_17 responses (125). Activated T cells release IL-23, which converts homeostatic T_H_17 cells to pathogenic T_H_17 cells, and play a major role in Crohn’s disease (244).

Crohn’s disease (CD) and ulcerative colitis (UC) are the most common chronic inflammatory bowel disorders (IBDs) (235, 236). The inflammatory infiltrate in IBDs is canonically characterized by activated T cells, macrophages, DCs, neutrophils, and T_H_17 cells (236). Basophils were identified in the inflamed mucosa of IBD patients that also expressed IL-33 (125). When activated by IL-3 and IL-33, basophils amplified T_H_17 cytokine expression in T cells. Basophils, but not mast cells, accumulated in inflamed CD and UC tissues compared to non-inflamed mucosa (237). No basophils were detected in colons of healthy control donors, indicating selective recruitment and/or survival of these cells at inflamed mucosal sites in patients with IBDs. The accumulation of basophils occurred in colons of untreated patients as well as in patients treated with 5-aminosalicylate acid or immunomodulators (e.g., glucocorticoids and/or immunosuppressive agents and/or biologics). Activated T cells infiltrate inflamed colons and are a major source of IL-3 (10) that may contribute to the infiltration and/or survival of basophils locally (238). Basophils increased IL-17 production and promoted the differentiation of IL-17^+^ cells. Collectively, these results demonstrate that basophils accumulate in the inflamed colon in patients with the two most frequent IBDs and may thus contribute to CD and UC pathogenesis. **Figure 6** schematically illustrates the potential mechanisms by which basophils, together with other immune cells, contribute to IBD.

## 10 Basophils in eosinophilic granulomatosis with polyangiitis

Eosinophilic granulomatosis with polyangiitis (EGPA) is a rare systemic disease characterized by eosinophilic asthma, sinus and pulmonary infiltrates, and eosinophil vasculitis (245). Lung biopsies are rarely done in EGPA and adequate animal models are not currently available. Therefore, the lung immunopathology of this disorder has not been carefully examined. Basophils were detected in four of five EGPA open lung biopsies (246), whereas no basophils were identified in seven control lung biopsies. Mast cell density was increased in EGPA patients compared to the control lungs. These preliminary data show that EGPA lung immunopathology includes infiltrates of eosinophils, basophils, and mast cells. Further studies appear necessary to identify possible interlinks between basophils and IgE and delineate the protective *versus* rather harmful effects of these conditions in EGPA.

Therapeutic management of EGPA is based on glucocorticoids alone and often in combination with immunosuppressive agents (247). Several observational studies have evaluated the role of omalizumab on maintenance therapy in EGPA (247–249). The results of these studies suggest that omalizumab may be clinically beneficial for EGPA patients improving asthma symptoms, lung function, and may have a glucocorticoid-sparing effect (247–249). There is the possibility that the effects of omalizumab in EGPA patients may be related, at least in part, to its effects on human basophils (250).

## 11 Basophils in eosinophilic esophagitis

Eosinophilic esophagitis (EoE) is a chronic, food-driven allergic disease characterized by esophageal eosinophilia that affects children and adults (251–253). The histopathological and clinical features of EoE have been attributed to overproduction of the type 2 cytokines IL-4, IL-5 and IL-13, which mediate profound alterations in the esophageal epithelium (254–256). The esophageal epithelium likely has an important role in the initiation of EoE *via* production of the epithelium-derived cytokines thymic stromal lymphopoietin (TSLP) and IL-33 (257, 258). EoE is associated with polymorphism in the gene that encodes TSLP in children (259, 260). In a mouse model, EoE-like disease developed independently of IgE, but was dependent on TSLP and basophils (257). Targeting TSLP or basophil depletion during the sensitization phase limited disease and improved established EoE-like disease. Interestingly, increased *TSLP* expression and basophil responses were demonstrated in esophageal biopsies of patients with EoE (257). Collectively, these results suggest that the TSLP-basophil axis contributes to the pathogenesis of EoE.

In another model of EoE-like disease, mice were epicutaneously sensitized with ovalbumin (OVA), followed by intranasal OVA challenge (258). This procedure promoted eosinophilic esophagitis, upregulation of Th2-like cytokines and the IL-33 receptor (ST2). *In vivo* basophil depletion or disruption of the IL-33-ST2 axis mitigated these features. These results suggest that basophils mediate experimental EoE through IL-33-ST2 interaction. These authors also found that pediatric patients with EoE have increased expression of *IL33* and *IL1RL1* (encoding ST2) in esophageal biopsies (258).

Taken together, these studies endorse the paradigm that epithelium-derived cytokines (i.e., TSLP and IL-33) play a role in the pathogenesis of EoE through the activation of basophils and the development of type 2 inflammatory milieu.

## 12 Concluding remarks and perspectives

Basophils are extremely rare cells, accounting for 1% or less of the circulating blood leukocytes, both in humans and mice. As a result, there was limited capacity to investigate the biology of these immune cells for several decades following their discovery in 1879 (261). However, advances during the past ~30 years have increased interest with compelling new evidence that they represent important effector cells in allergic inflammation (1, 64, 81, 82) and exert a protective role in parasitic infections (66–68). The development of new murine genetic tools and different models of inflammation has also generated novel insight into the potential contribution of basophils to an increasing spectrum of diseases. In particular, basophils and their mediators are now implicated as important participants in pathophysiologic conditions never before considered, including MI (70), kidney fibrosis (71), several autoimmune disorders (76, 77, 126), different cancers (72, 73, 75), COPD (62), and COVID-19 (230–232, 234).

In several pathological conditions, such as kidney fibrosis (71), autoimmune disorders (76, 77, 125, 126), some cancers (72, 73), COPD (62), IgG4-RD (208), IBD (125, 237), and EoE (257, 258) basophils and their mediators play a harmful role. In other inflammatory disorders, such as MI (70), certain cancers (154) (75), and COVID-19 (230–232, 234), basophils appear to play a protective role. The dichotomous pathogenic role of basophils is intriguing and will undoubtedly be the subject of future investigations. There is the possibility that, like mast cells (262–266), macrophages (104, 132, 267, 268), neutrophils (269–272), and eosinophils (273, 274), subpopulations of basophils may also exist. In this regard, distinct phenotypic and functional basophil subpopulations have been described in human peripheral blood (275). Moreover, it has already been demonstrated that tissue-resident basophils differ from circulating basophils in mice (276) and possibly in humans. Finally, basophils might possess a high degree of plasticity and can modify their phenotype and functional characteristics when exposed to different local environments. Whatever the case, the possible existence of basophil subpopulations and the disease-specific heterogeneity of these cells need to be thoroughly and accurately explored in both humans and mice by novel analytical tools (e.g., single-cell RNA seq, CyTOF).

Finally, several biologics have been approved for the treatment of severe allergic disorders and are showing remarkable efficacy (218). Those designed primarily to target mast cells, eosinophils, and Th2 cells (e.g., omalizumab, mepolizumab, benralizumab and dupilumab) also target human basophils and/or their products (250, 277). Thus, there is the possibility that these biologics could prove efficacious in helping to combat other unsuspected conditions/diseases (e.g., cancer, autoimmunity, fibrosis) where basophils are recently implicated. In contrast, with mounting evidence that basophils and their mediators also play critical homeostatic and protective roles (70, 75, 226, 230–232, 234), caution may be warranted when these therapeutic interventions are used.

## Author contributions

RP, GM, JS, GV drafted the manuscript and interpreted data; RP, SL, GM, AS, AP, JS, GV edited the manuscript. All authors contributed to the article and approved the submitted version.
